# A *L2HGDH* initiator methionine codon mutation in a Yorkshire terrier with L-2-hydroxyglutaric aciduria

**DOI:** 10.1186/1746-6148-8-124

**Published:** 2012-07-26

**Authors:** Fabiana HG Farias, Rong Zeng, Gary S Johnson, G D Shelton, Dominique Paquette, Dennis P O’Brien

**Affiliations:** 1 Department of Veterinary Pathobiology, University of Missouri, Columbia, USA; 2 Department of Pathology, University of California at San Diego, La Jolla, USA; 3 Centre Veterinaire DMV, Montreal, Canada; 4 Department of Veterinary Medicine and Surgery, University of Missouri, Columbia, USA

**Keywords:** L-2-hydroxyglutaric aciduria, *L2HGDH*, Yorkshire terrier, Initiator methionine codon

## Abstract

**Background:**

L-2-hydroxyglutaric aciduria is a metabolic repair deficiency characterized by elevated levels of L-2-hydroxyglutaric acid in urine, blood and cerebrospinal fluid. Neurological signs associated with the disease in humans and dogs include seizures, ataxia and dementia.

**Case presentation:**

Here we describe an 8 month old Yorkshire terrier that presented with episodes of hyperactivity and aggressive behavior. Between episodes, the dog’s behavior and neurologic examinations were normal. A T2 weighted MRI of the brain showed diffuse grey matter hyperintensity and a urine metabolite screen showed elevated 2-hydroxyglutaric acid. We sequenced all 10 exons and intron-exon borders of *L2HGDH* from the affected dog and identified a homozygous A to G transition in the initiator methionine codon. The first inframe methionine is at p.M183 which is past the mitochondrial targeting domain of the protein. Initiation of translation at p.M183 would encode an N-terminal truncated protein unlikely to be functional.

**Conclusions:**

We have identified a mutation in the initiation codon of *L2HGDH* that is likely to result in a non-functional gene. The Yorkshire terrier could serve as an animal model to understand the pathogenesis of L-2-hydroxyglutaric aciduria and to evaluate potential therapies.

## Background

L-2 hydroxyglutaric aciduria (L-2-HGA) was first described in 1980 in a young boy with psychomotor retardation and musculoskeletal dystrophy [1]. This rare autosomal recessive inherited disease is characterized by an elevated concentration of L-2-hydroxyglutaric acid in plasma, cerebrospinal fluid, and urine [1]. In human patients, the clinical features are mild psychomotor delay, followed by progressive cerebellar ataxia, dysarthria, moderate to severe mental deterioration and in some cases seizures [2,3]. Magnetic resonance imaging (MRI) of L-2-HGA patients shows abnormal signal in the peripheral subcortical white matter, basal ganglia and dentate nuclei. Also, cerebellar atrophy is present. The distribution of signal abnormalities in the MRI of L-2-HGA patients is distinct and differentiates it from other diseases [4,5].

In 2004, mutations in *L2HGDH* were shown to cause human L-2-HGA [3,6]. *L2HGDH* is comprised of 10 exons which encode a protein of 463 amino acids that contains a N-terminal mitochondrial targeting sequence and C-terminal amino acid sequence homology to FAD-dependent oxidoreductases [6]. L-2-hydroxyglutarate dehydrogenase is bound to mitochondrial membranes and catalyzes the conversion of L-2-hydroxyglutarate to α-ketoglutarate [6].

L-2-HGA was described in Staffordshire bull terriers in 2003 [7]. These dogs presented with ataxia, dementia, tremors and seizures, and they showed MRI changes similar to the human disease. L-2-hydroxyglutaric acid levels were elevated in urine, cerebrospinal fluid and plasma from affected dogs. In 2007, Penderis et al. [8] identified a 2 bp substitution in exon 10 of *L2HGDH* predicting a two amino acid substitution in Staffordshire bull terriers affected with L-2-HGA. A case of L-2-HGA was also reported in a West Highland white terrier with clinical and MRI characteristics of the disease [9], but no molecular genetic cause was determined. In this report, we describe the clinical features and the likely molecular genetic cause of L-2-HGA in a Yorkshire terrier.

## Case presentation

**Figure 1:**
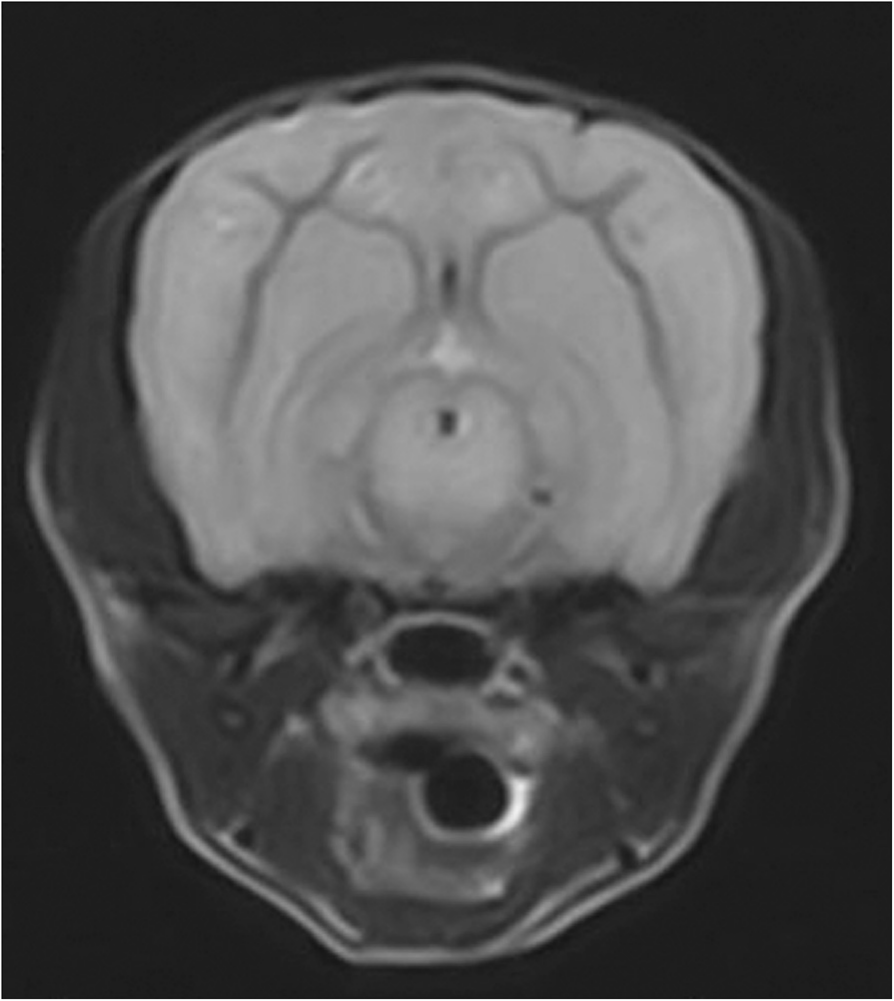
**Transverse T2-weighted image (TR = 3000, TE = 90) through the midbrain of the Yorkshire terrier with L-2-HGA.** The gray matter of the cerebrum and brainstem shows the symmetric hyperintensity characteristic of the disease [7,9].

An 8 month old neutered male Yorkshire terrier was presented with episodes of hyperactivity and aggressive behavior. The episodes would last for about 40 minutes and then the dog behaved normally between episodes. No abnormalities were found on neurologic examination. Cerebrospinal fluid analysis showed a mild mononuclear pleocytosis (WBC 23/ul {N<8} with 92% mononuclear cells and 8% small lymphocytes) and normal protein (7 mg/dl {N<36}). An MRI of the brain (Figure 1) demonstrated hyperintensity of the gray matter of the thalamus, cerebral cortex and cerebellum on T2 weighted images similar to previously described MRIs from Staffordshire bull terriers and a West Highland white terrier with L-2-HGA [7,9]. No abnormalities were apparent on FLAIR, gradient echo or T1 weighted images with or without gadolinium contrast. Urine organic acid levels were quantified by gas chromatography-mass spectroscopy as previously described [10], and showed an elevated concentration of hydroxyglutaric acid (1,743 mmol/mol creatinine {N = 0.6–5.7}). Plasma amino acid levels were also quantified and showed elevated lysine (600 μmol/L {N = 145–201}). A lysine restricted diet was developed, but it was not palatable so the dog was placed on a commercial protein restricted diet^1^. The dog was also started on phenobarbital at 2.5 mg/kg. The frequency of the episodes decreased with treatment, and the dog showed no progression of signs over 6 months.

**Figure 2:**
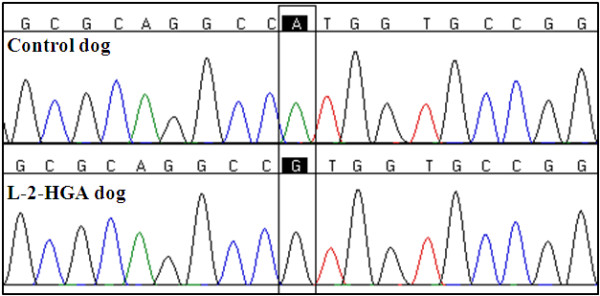
**Partial nucleotide sequence of *L2HGDH *** **exon 1 from a control dog and the Yorkshire terrier with L-2-HGA illustrating the** *** L2HGDH *:c.1A>G mutation.**

Because MRI findings and urinary organic acid profiles were consistent with a diagnosis of L-2-HGA, *L2HGDH* became the most likely candidate gene to harbor the mutation responsible for the 2-HGA in this dog. DNA was extracted from an EDTA blood sample using a previously described method [11]. First, we eliminated the presence of the mutation identified in Staffordshire bull terriers [8]. To investigate whether there were other sequence variants in the affected dog’s *L2HGDH*, we sequenced all 10 exons and intron–exon junctions as previously described [12]. Primer sequences are provided in Additional file 1. Comparison of the sequencing data from all 10 exons of canine *L2HGDH* from the affected dog to the published canine sequence (Ensembl Gene ID ENSCAFG0000001437) revealed a homozygous *c.1A>G* substitution (Figure 2). This nucleotide substitution changes the initiation methionine codon, which is predicted to alter the translation start site [13].

We genotyped 6 unaffected Yorkshire terriers and 97 dogs from other breeds at *L2HGDH:c.1* by PCR-RFLP with PCR primers 5′-GCGCTCGATTGGCCCTTG-3′ and 5′-GCAGGCCAGCGGCTACTCAC-3′ which produced a 298 bp amplicon. The wild type allele was hydrolyzed by restriction enzyme *Nco*I into fragments of 157 and 141 bp; whereas, the mutant allele lacked the *Nco*I restriction site. All 103 normal dogs were homozygous for the wild type A allele.

*L2HGDH* encodes a FAD-dependent L-2-hydroxyglutarate dehydrogenase that is bound to mitochondrial membranes and converts L-2-hydroxyglutarate to α-ketoglutarate [6]. L-2-hydroxyglutarate is endogenously produced but is not an intermediate of any currently recognized mammalian metabolic pathway. Instead, L-2-hydroxyglutarate is formed because mitochondrial L-malate dehydrogenase is not completely specific for oxaloacetate, its primary substrate. This enzyme can also reduce α-ketoglutarate to L-2-hydroxyglutarate as a side reaction [14]. L-2-hydroxyglutarate dehydrogenase prevents the accumulation of L-2-hydroxyglutarate by FAD-dependent oxidation back to α-ketoglutarate. Thus, L-2-HGA is considered to be a deficiency of ‘metabolite repair’ [6,15,16].

The deleterious consequence of L-2-hydroxyglutarate dehydrogenase deficiency is the accumulation of L-2-hydroxyglutarate. L-2-hydroxyglutarate induces oxidative stress and inhibits mitochondrial creatine kinase in the cerebellum [17,18]. Also, L-2-hydroxyglutarate is a structural analog of glutamate, and thus a potential inhibitor of the many biologic processes that involve this key metabolite and neurotransmitter [15]. The exact mechanism by which accumulation of L-2-hydroxyglutaric acid causes brain injury is still unknown.

Since the identification of the first human *L2HGDH* mutations [3,6], about 70 L-2-HGA-causing mutations have been reported [19]. Among these are two that occur in the initiator codon (*c.1A>C* and *c.1A>G*), similar to the canine *L2HGDH*:c*.1A>G* mutation reported here. The canine initiator codon mutation was absent from 103 unaffected dogs. Mutations in initiator codons require the use of an alternative downstream methionine codon [13]. The next potential translational start site in the canine *L2HGDH* is out of frame and would encode a peptide of only 4 amino acids before it reaches a stop codon. The first inframe methionine codon is located 183 codons downstream from the original start codon, which if used would encode a protein without the N-terminal mitochondrial targeting domain [15]. Thus, the mutant *L2HGDH* in the Yorkshire terrier with L-2-HGA is unlikely to be functional.

The only clinical signs shown by the Yorkshire terrier were episodes of hyperactivity and aggression. At the time of this report, the dog has not shown any dementia, ataxia or generalized seizures as has been described in human patients and dogs with L-2-HGA from other breeds. Unless there is an unidentified splice variant that provides an alternative initiator methionine codon, the milder phenotype is unlikely to be due to residual enzyme activity. It could reflect a response to therapy or the younger age of the Yorkshire terrier. The decreased severity may have been due to the altered diet or to the phenobarbital therapy which would suggest that these episodes were complex partial seizures, possibly with post-ictal behavior changes.

## Conclusion

We described a previously unreported mutation in *L2HGDH * in a Yorkshire terrier with L-2-HGA. This mutation is predicted to result in a non-functional gene. We recently learned that another group has independently found the same mutation in two European Yorkshire terriers with L-2-HGA [20]. The occurrence of L-2-HGA in two European Yorkshire terriers with an identical mutation is additional evidence that the *L2HGDH*:*c.1A>G* transition is the cause of L-2-HGA in this breed. The similarities between canine L-2-HGA and the human disease suggest that the canine disease could be a useful model to understand the pathogenesis of the disease and to evaluate potential therapies.

## Endnotes

^1^L/D, Hill’s Pet Nutrition, Inc., Topeka, KS, USA.

## Authors’ contribution

FHGF analyzed sequences of *L2HGDH*, genotyped normal dogs and drafted the manuscript and submitted the manuscript for publication. RZ genotyped the Yorkshire terrier for the mutation identified in Staffordshire bull terriers. GSJ designed experiments and helped to draft the manuscript. Analysis of organic acids in the urine was performed by GDS. DP performed the clinical and neurological examinations and generated the MRI. DPO contributed to the clinical assessment and helped to draft the manuscript. All authors contributed to the preparation of the manuscript. All authors read and approved the final manuscript.

## Supplementary Material

Additional file 1 **Primer sequences for amplification of canine *L2HGDH.***
Click here for file
