# Supplementary material for: A L2HGDH initiator methionine codon mutation in a Yorkshire terrier with L-2-hydroxyglutaric aciduria
Source: BMC Vet Res. 2012 Jul 26;8:124. doi: 10.1186/1746-6148-8-124 (PMC3461439; doi:10.1186/1746-6148-8-124)
Supplement: Additional file 1 — Primer sequences for amplification of canine L2HGDH. [file 1746-6148-8-124-S1.doc]

**Additional file 1.** Primer sequences for amplification of canine *L2HGDH*.

| **Target** | **Forward Primer Sequences / Reverse Primer Sequence** | **Amplicon**  **Length (bp)** |
| --- | --- | --- |
| **Exon 1** | **CGCGCTCGATTGGCCCTTGA / AGCAGTAGTAGCACAGGCGACGA** | **322** |
| **Exon 2** | **ACAAATTAATAGTTTGATCGTCC / TACTCAGGTTAGGCCCAAA** | **260** |
| **Exon 3** | **CACTTTGTAGGTTTTCTATGCAC / AGTTCTAAGTCAAGGCCCTA** | **247** |
| **Exon 4** | **CATGTGATTATTTATTTTTATTGAC / TGTAAATTTACCCTCAGC** | **321** |
| **Exon 5** | **ATTCAGTCCCAAGTAAAATTTATTGA / AGCCCCTGGTGGCCCT** | **354** |
| **Exon 6** | **TAGTGTATCAAAAATGTAGGC / GGACATAGAAAACAACAT** | **201** |
| **Exon 7** | **CCCCTTGACCTGTGTTTCC / GCCAGATGCTCAATCGTT** | **328** |
| **Exon 8** | **ATTGGAAGAAAGAGTTTTATCAGC / ACCCAATAAACAGAACATCAAGA** | **337** |
| **Exon 9** | **CTATGTTTCTTTGATGCCAC / TACAAAGATTCAGTAGGCAC** | **353** |
| **Exon 10** | **ACAATTTGTAAAGTGCCCAA / CATTTTCCTTAAAGAATGCAA** | **336** |
